# Validation of Simple Methods to Select a Suitable Nostril for Nasotracheal Intubation

**DOI:** 10.1155/2018/4910653

**Published:** 2018-08-01

**Authors:** Cattleya Thongrong, Pattramon Thaisiam, Pornthep Kasemsiri

**Affiliations:** ^1^Department of Anesthesiology, Faculty of Medicine, Khon Kaen University, Khon Kaen, Thailand; ^2^Division of Skull Base Surgery, Department of Otorhinolaryngology, Faculty of Medicine, Khon Kaen University, Khon Kaen, Thailand

## Abstract

**Background:**

Nasotracheal intubation is a blind procedure that may lead to complications; therefore, several tests were introduced to assess a suitable nostril for nasotracheal intubation. However, the value of simple tests in clinical practice was insufficient to evaluate.

**Method:**

A diagnostic prospective study was conducted in 42 patients, ASA classes I–III, undergoing surgery requiring nasotracheal intubation for general anesthesia. Two simple methods for assessing the patency of nostrils were investigated. Firstly, the occlusion test was evaluated by asking for the patient's own assessment of nasal airflow during occlusion of each contralateral nostril while in a sitting posture. Secondly, patients breathed onto a spatula held 1 cm below the nostrils while in a sitting posture. All patients were assessed using these two simple tests. Nasal endoscopic examination of each patient was used as a gold standard.

**Results:**

The diagnostic value of the occlusion test (sensitivity of 91.7%, specificity of 61.1%, PPV of 75.9%, NPV of 84.6%, LR+ of 2.36, and LR− of 0.14) seemed better than that of the spatula test (sensitivity of 95.8%, specificity of 25.0%, PPV of 63.0%, NPV of 81.8%, LR+ of 1.28, and LR− of 0.17). When both tests were combined in series, the diagnostic value increased (sensitivity of 87.9%, specificity of 70.8%, PPV of 80.1%, NPV of 81.4%, LR+ of 3.01, and LR− of 0.17).

**Conclusion and Recommendations:**

The simple occlusion test is more useful than the spatula test. However, combining the results from both tests in series helped to improve the diagnostic value for selecting a suitable nostril for nasotracheal intubation.

## 1. Introduction

Nasotracheal intubation is one of the most common methods for established airway management in patients undergoing surgeries, especially in the head and neck region. However, nasotracheal intubation is a blind procedure that may lead to complications such as epistaxis, avulsion of the middle and inferior turbinate [1–4], and tearing of the nasal septum. Numerous methods have been proposed to reduce these complications. Recommended methods include application of adrenaline [5], use of a smaller tracheal tube, use of a gastric tube or a suction tube for guidance [6, 7], softening the tracheal tube with warm water [8], and redesigning the nasotracheal tube tip [9–12].

Furthermore, the patency of the nostril is a very important factor in reducing injury to nasal passages. Recently, rhinomanometry has been introduced to evaluate the patency of nostrils [13–15]. However, this method is not commonly used in clinical practice because it is not simple and requires special equipment; thus, simpler techniques are preferred. Proposed simple methods include examining the pattern of condensation from expired breath on a spatula [16], inspecting the caudal end of the nasal septum [16], palpating the airflow from each nasal passage [17], and conducting a preanesthetic interview during which the patient is asked which one they perceive to be the clearer nostril [17, 18]. However, the diagnostic value of these simple techniques has not been studied adequately. This study was developed to compare the reliability of simple clinical assessment of the patency of nostrils with results from nasal endoscopic examination.

## 2. Materials and Methods

The Human Research Ethics Committee of Khon Kaen University approved this study protocol (HE601144), and written informed consent was obtained from each patient. All patients undergoing elective surgery and with the requirement of nasotracheal intubation in Srinagarind Hospital, Khon Kaen, Thailand, from July 1, 2017 to December 1, 2017, were enrolled. Patients of either gender aged less than 18 years or more than 75 years were excluded. Also excluded were patients conforming to physical status classification IV (American Society of Anesthesiologists), those with a history of nasal fracture or previous nasal surgery, those with a history of sinusitis or nasal tumor, and those with underlying blood dyscrasia.

We used two simple methods to assess the patency of nostrils. Firstly, the occlusion test, which was administered by asking for the patient's own assessment of nasal airflow during occlusion of the contralateral nostril while in a sitting posture. Nostrils were classified as left or right nostril clearer, or both equally clear. Secondly, patients breathed onto a spatula held 1 cm below the nostrils while in a sitting posture. The patterns of condensation on the spatula were used to determine whether or not one nostril was clearer than the other. If the area of condensation on one side had a diameter of 1 cm greater than that on the other, then that side was regarded as the clearer. These simple methods were assessed by one anesthesiologist. Subsequently, the nostrils were assessed with a 2.7 mm 0-degree nasal endoscope by one otorhinolaryngologist who did not know the previous anesthesiologist's results. The morphology of each nostril was graded in terms of nasal septum deviation (I = deviation 0–24%, II = deviation 25–49%, III = deviation 50–74%, and IV = deviation ≥75%) and inferior hypertrophy (I = nostril occluded by 0–24%, II = nostril occluded by 25–49%, III = nostril occluded by 50–74%, and IV nostril occluded by ≥75%). The standard left-facing bevel nasotracheal tube was prepared with thermosoftening and lubrication before it was inserted into the nostril that was regarded as more patent according to the nasal endoscopic finding. The number of attempts at intubation and severity of epistaxis were recorded.

Qualitative variables are presented as proportions, whereas quantitative variables are expressed as means and standard deviation. The nasal endoscopic examination was considered as the “gold standard” test for determining the patency of the nostril. The diagnostic value of each simple method was calculated using STATA version 10.0. Furthermore, we combined the results of the two simple tests in series and in parallel to see if this would yield improved diagnostic value. Sensitivity and specificity values are given, as well as predictive values and likelihood ratios, all with associated 95% confidence intervals.

## 3. Results

Patient characteristics are shown in Table 1. The majority of patients were undergoing maxillofacial surgery. Most patients had grade I deviation of the nasal septum, especially of the right side (Table 2). Only two patients had grade IV deviation of the nasal septum. Inferior turbinate hypertrophy is another troublesome factor for nasotracheal intubation. Almost half of patients had grade II enlargement of the inferior turbinate. The nasal endoscopic examination guided the choice of nostril for intubation. Nasotracheal intubation was successful on the first attempt for 33 of 42 patients. More than two attempts were required for one patient who presented with grade III hypertrophy of the inferior turbinate on both sides. Epistaxis was observed in 69.05% of cases, with mild bleeding. This epistaxis complication was not statistically significantly different between the right and left nostril (*p* value > 0.05) (Table 3).

The diagnostic value of the simple tests was investigated (Table 4). The occlusion test seemed appropriate for assessing the suitable nostril for intubation due to its high sensitivity (91.7%) and fair specificity (61.1%). Combining the test results in series provided a better diagnostic value with good stratified sensitivity (87.9%) and higher specificity (70.8%).

## 4. Discussion

Assessment of nostrils before nasotracheal intubation is very important to reduce subsequent complications. Although rhinomanometry and endoscopy are more reliable than simple methods, they are not convenient for use in common practice. Simple methods are preferable to guide the choice of nostril to intubate. Smith and Reid [17] studied the accuracy of palpating the passage of air and asking for the patient's own assessment of nasal airflow. They used nasal endoscopic examination as the gold standard. They reported no significant difference between the overall diagnostic success rates of the two tests (44% and 47%, resp.). Unfortunately, they found the tests had a diagnostic failure rate of approximately 45% when an abnormal nostril was present. Thus, they recommended that anesthesiologists should not rely on the two tests investigated. However, we reassessed the patient's own opinion of nasal airflow method. Its sensitivity was 91.7%, specificity was 61.1%, positive predictive value was 75.9, and positive likelihood ratio was 2.36.

For improved diagnostic value, the occlusion test was combined with the spatula test because it was a simple method with high sensitivity (95.8%). We investigated the diagnostic value of combining the results from the two tests in series and in parallel. When in series, both tests must be positive for an overall positive result to be accepted. Tests in series yielded a lower overall sensitivity than either alone, but the overall specificity was greater than for either alone. On the other hand, when in parallel, a positive result in either test should be taken as an overall positive result. Parallel interpretation of tests yielded greater overall sensitivity than either alone, but the overall specificity was less than for either alone. Therefore, the series approach was regarded as better than the parallel approach. In series, the tests provided a sensitivity of 87.9%, specificity of 70.8%, positive predictive value of 80.1, and positive likelihood ratio of 3.01. We therefore recommend use of results from both tests in series for guiding selection of the suitable nostril for nasotracheal intubation.

There have been several reports concerning selection of a nostril for nasotracheal intubation. In a randomized controlled trial, Boku et al. [18] found that nasal intubation via the right nostril produced less bleeding than did using the left nostril (*p*=0.0006). These outcomes were similar to the study of Sanuki et al. [19], who reported that epistaxis during nasotracheal intubation was more frequent and severe when the left nostril was used. Nonetheless, Coe and Human [20] had demonstrated similar frequencies of epistaxis in both nostrils. This result was similar with our study. We found no statistically significant different epistaxis between both nostrils (*p* > 0.05) although we used the standard left-facing beveled nasotracheal tube. We gently inserted the well-lubricated nasotracheal tube with bevel of the tube against nasal septum or floor of nose for reduced injury to inferior turbinate. However, our small sample size was limited to indicate difference in this aspect. Furthermore, the dominant hand nature of the anesthesiologist is the other factor that may affect epistaxis complication from nasotracheal intubation. Usually, an anesthesiologist uses the dominant hand for the insertion tube; therefore, right-handed nature may prefer right nostril, whereas left-handed nature may prefer left nostril. We concern that the left-facing bevel of the tube may cause injury to inferior turbinate when introducing the tube through the left nostril due to the bevel of the tube against the turbinate. On the other hand, the bevel of the tube against the nasal septum when using the right nostril. It may help to reduce the chance of epistaxis on the right side. However, nasotracheal intubation on the left nostril is not a big deal. We recommend introducing the tube with some rotation to present the bevel against the floor of nose for avoiding injury to inferior turbinate. After the tip of the tube had been passed from the end of inferior turbinate to choana, the tube was rotated into the normal position for passing through the pharynx.

Accordingly, nasotracheal intubation is a blind procedure. The nasal examination will lead to a better understanding of the patency of each nostril and lead to reduction in complications. We urge anesthetists to assess the state of each nostril prior to intubation of their patients.

## 5. Conclusion

The simple occlusion test was better than the spatula test in guiding the choice of nostril for intubation. However, the combination of results from both tests in series provided the most reliable guidance.

## Figures and Tables

**Table 1:** Demographic data.

Characteristics	Number	95% CI
*Gender*		
Male (%)	14 (33.33)	21.01–48.45
Female (%)	28 (66.77)	51.55–78.99
Age (yr, mean)	47.98 (16.73)	42.76–53.19
Weight (kg, mean)	54.81 (11.51)	51.22–58.40
Height (cm, mean)	157.81 (7.95)	155.33–160.29
BMI	21.90 (3.80)	20.72–23.08
*ASA class*		
I (%)	25 (59.52)	44.49–72.96
II (%)	17 (40.78)	27.04–55.51
III (%)	0	0
*Underlying disease*		
Diabetes mellitus	5 (11.90)	5.19–25.00
Hypertension	7 (16.67)	8.32–30.60
Dyslipidemia	1 (2.38)	0.42–12.32
*Operation*		
Endoscopic transoral thyroidectomy	6 (14.29)	6.72–27.84
Wide excision with or without soft tissue reconstruction	13 (30.95)	19.07–46.03
Maxillofacial surgery	23 (54.76)	39.95–68.78
*Attempts at intubation*		
Once	33 (78.57)	64.06–88.29
Twice	8 (19.05)	9.98–33.30
More than twice	1 (2.38)	0.42–12.32
*Severity of epistaxis*		
No bleeding	13 (30.95)	19.07–46.03
Minimal bleeding	29 (69.05)	53.97–80.93

**Table 2:** Nasal endoscopic finding.

Nasal endoscopic finding: deviated nasal septum	Right nostril (%)	Left nostril (%)
Grade I: 0–24%	29 (69.05)	19 (45.24)
	(95% CI: 53.97–80.93)	(95% CI: 31.22–60.05)
Grade II: 25–49%	8 (19.05)	9 (21.43)
	(95% CI: 9.98–33.30)	(95% CI: 11.71–35.94)
Grade III: 50–74%	4 (9.52)	13 (30.95)
	(95% CI: 3.77–22.07)	(95% CI: 19.07–46.03)
Grade IV: ≥75%	1 (2.38)	1 (2.38)
	(95% CI: 0.42–12.32)	(95% CI: 0.42–12.32)

*Inferior turbinate hypertrophy*		
Grade I: 0–24%;	4 (9.52)	5 (11.90)
	(95% CI: 3.77–22.07)	(95% CI: 5.19–25.00)
Grade II: 25–49%	20 (47.62)	28 (66.67)
	(95% CI: 33.36–62.28)	(95% CI: 51.55–78.99)
Grade III: 50–74%	16 (38.10)	8 (19.05)
	(95% CI: 25.00–53.19)	(95% CI: 9.98–33.30)
Grade IV: ≥75%	2 (4.76)	1 (2.38)
	(95% CI: 1.32–15.79)	(95% CI: 0.42–12.32)

**Table 3:** Comparison of epistaxis complication between right and left nasotracheal intubation.

Nasal endoscopic finding	Number	Nasotracheal intubation						
		Side	One attempt		Two attempts		More than two attempts	
			No bleeding	Minimal bleeding	No bleeding	Minimal bleeding	No bleeding	Minimal bleeding
More patent on the right side	22	Right	5 (22.7)^†^	13 (59.1)^†^	0	4 (18.18)^††^	0	0
			(95% CI: 10.1–43.4)	(95% CI: 38.7–76.7)		(95% CI: 7.3–38.5)		

Equal patent on both sides	6	Right	3 (50.0)^†^	2 (33.3)^†^	0	0	0	0
			(95% CI: 18.8–81.2)	(95% CI: 9.7–70.0)				
		Left	0	0	0	0	0	1 (16.7)^††^
								(95% CI: 0.3–56.4)

More patent on the left side	14	Left	5 (35.7)^†^	5 (35.7)^†^	0	4 (28.6)^††^	0	0
			(95% CI: 16.3–61.2)	(95% CI: 16.3–61.2)		(95% CI: 11.7–54.7)		

^†^No statistically significant difference of no epistaxis event (*p*=0.84) and minimal epistaxis (*p*=0.15) between both nostrils at one attempt of intubation. ^††^No statistically significant difference of minimal epistaxis between both nostrils at two attempts of intubation (*p*=0.34) and more than two attempts of intubation (*p*=0.16).

**Table 4:** Diagnostic value of the occlusion test, spatula test, and two tests combined (series test and parallel test).

	Sensitivity	Specificity	Predictive value		Likelihood ratio	
			Positive	Negative	Positive	Negative
Occlusion test	91.7	61.1	75.9	84.6	2.36	0.14
	(95% CI: 80.0–97.7)	(95% CI: 43.5–76.9)	(95% CI: 62.8–86.1)	(95% CI: 65.1–95.6)	(95% CI: 1.55–3.58)	(95% CI: 0.05–0.36)

Spatula test	95.8	25.0	63.0	81.8	1.28	0.17
	(95% CI: 85.7–99.5)	(95% CI: 12.1–42.2)	(95% CI: 50.9–74.0)	(95% CI: 48.2–97.7)	(95% CI: 1.05–1.56)	(95% CI: 0.04–0.73)

Series test	87.9	70.8	80.1	81.4	3.01	0.17
	(95% CI: 78.6–97.1)	(95% CI: 56.0–85.7)	(95% CI: 68.1–92)	(95% CI: 69.5–93.3)	(95% CI: 1.82–4.99)	(95% CI: 0.08–0.38)

Parallel test	99.7	15.3	61.1	97.0	1.18	NA
	(95% CI: 98.0–100)	(95% CI: 3.5–27.0)	(95% CI: 50.2–71.9)	(95% CI: 83.5–100)	(95% CI: 1.02–1.36)	

## Data Availability

The data used to support the findings of this study are available from the corresponding author upon request.
